# Optimising gravimetric method interpretation using population-specific blood densities among Indonesian pregnant women: A cross-sectional study

**DOI:** 10.1371/journal.pone.0339391

**Published:** 2026-01-08

**Authors:** Nelil Mudarris, Indrayani Indrayani, Wisnu Arfian A. Sudjarwo, Dewi Anggraini, Dyan Oktaviany, Devendra Raj Singh, Galuh Nugraheni, Aminah Syafa’ah

**Affiliations:** 1 Department of Midwifery, Universitas Bima Sakapenta, Bogor, Indonesia; 2 School of Human and Health Sciences, University of Huddersfield, Huddersfield, United Kingdom; 3 Department of Midwifery, Akademi Kebidanan Bina Husada Tangerang, Banten, Indonesia; 4 Institute of Biophysics, Department of Natural Science and Sustainable Resources, BOKU University, Vienna, Austria; 5 Department of Statistics, Faculty of Mathematics and Natural Sciences, Universitas Lambung Mangkurat, South Kalimantan, Indonesia; 6 Department of Midwifery, Politeknik Bakti Asih Purwakarta, West Java, Indonesia; Kasr Alainy Medical School, Cairo University, EGYPT

## Abstract

**Background:**

The diagnosis of postpartum haemorrhage (PPH) depends heavily on accurate and timely blood loss assessment, yet no gold standard assessment method is available. Gravimetric methods are recommended for low-resource settings, but they require an accurate blood density value for interpretation. Currently, there are no data on the blood density of pregnant or labour women.

**Objective:**

This study measured the blood density of Indonesian pregnant women to improve the reliability of the gravimetric results in estimating postpartum blood loss.

**Methods:**

This cross-sectional study was conducted at a Private Midwifery Service Clinic in Bogor, Indonesia, involving 44 pregnant women who were at 37 weeks of gestation or later. During antenatal visits, 5 ml venous blood samples were collected, with women experiencing high-risk pregnancies being excluded. Descriptive statistics summarised the characteristics of the participants. Spearman’s rank-order correlation was used to assess associations between study variables, and the Kruskal–Wallis test evaluated group differences.

**Results:**

The mean blood density was 1.060 ± 0.080 g/ml (range: 0.860–1.194 g/ml), with stable indoor and outdoor temperatures of 27 °C (SD = 1.13 °C and 1.22 °C, respectively). No significant correlations were found between blood density and maternal age, pre-pregnancy BMI, or gestational weight gain (p > 0.05). Kruskal–Wallis tests also revealed no significant differences across these variables (p > 0.05).

**Conclusion:**

Blood density among Indonesian pregnant women corresponds with established human reference values, supporting the use of population-specific blood density values to improve gravimetric blood loss estimation accuracy, thus facilitating early detection and prompt intervention of PPH. The observed variability suggests that factors other than demographics, maternal anthropometry, and the environment may affect blood density, necessitating further investigation in larger multisite studies.

This study was reported in accordance with the STROBE checklist; the completed checklist is available as Supporting Information (S1 File).

This study employed a cross-sectional design to measure blood densities of pregnant women at a single point in time. Participants were recruited during antenatal visits at Dyan’s Private Midwifery-Service Clinic, Gunung Putri, Bogor, West Java, Indonesia. The health facility is a primary-level facility led by midwives and serves as the community’s first point of contact for maternal and child health services. The health facility offers routine maternal health services, including antenatal care, childbirth assistance, postpartum follow-up, and family planning. As a primary-level facility, this health facility only provided care for women with low-risk pregnancies following the midwife’s authority, while women with high-risk pregnancies or obstetrical/pathological conditions were referred to referral or obstetrician-led care facilities. The facility, situated in Gunung Putri, an industrial area adjacent to the capital city (Jakarta), serves a stable yet demographically diverse population drawn from various provinces and socioeconomic backgrounds, offering heterogeneous and broadly representative of Indonesia’s diverse demographics. Although not fully representative of rural or coastal populations, the site offers a scientifically appropriate environment for studying blood density among pregnant Indonesian women. Prior to the commencement of the study, the participating facility provided permission for data collection. Data collection began on 8^th^ January 2025 and ended on 21^st^ April 2025.

This study included pregnant women 37 weeks pregnant or older (based on the first day of the last menstrual cycle). To measure blood density, 5 ml venous blood samples were collected from all eligible participants. Pregnant women at high risk of pregnancy complications were not eligible to participate in the study. To ensure appropriate representation of the target population in this study, the sample size was determined using the Slovin formula by considering the total number of antenatal visits at Dyan’s Private Midwifery Service Clinic between November 2023 and October 2024, which was 2,576. With an error tolerance of 0.15, a sample of 44 pregnant women was deemed sufficient to balance statistical precision with study feasibility. Given the exploratory, cross-sectional nature of the work, which focused on estimating blood density rather than group comparisons or hypothesis testing, this margin of error provided an acceptable level of precision. Accordingly, a sample size of 44 was adequate to support the optimisation of the gravimetric method for estimating postpartum blood loss. A consecutive sampling method was used to recruit participants. All pregnant women at the study site who met the predefined inclusion criteria were invited to participate in the study. Recruitment continued until the target sample size was achieved. As a preliminary cross-sectional study to estimate blood density and not population-level prevalence, consecutive sampling was considered suitable. All eligible participants were enrolled during the study period to minimise selection bias.

## Background

Postpartum haemorrhage (PPH), which affects millions of women every year and accounts for 25% of maternal deaths worldwide [1] and 30% of maternal deaths in Indonesia [2], is a significant global health concern. It is imperative to detect PPH, a blood loss of 500 ml or more within 24 hours after birth [3], early so that effective treatment can be initiated, and the potential life-threatening outcome minimised [4]. PPH diagnosis relies heavily on timely and accurate blood loss assessment. Despite its importance, no universally accepted gold standard for measuring postpartum blood loss exists [5]. It is crucial to address the challenges associated with blood loss assessment to reduce preventable maternal deaths and ensure timely PPH management.

The gravimetric method is recommended for assessing postpartum blood loss in low-resource settings because it is objective, simple, and time-efficient [6,7], with a significant correlation with colourimetric haemoglobin measurements [6]. In this method, all blood-soaked materials (e.g., sponges, swabs, and pads) are weighed before and after use [8] (including expelled blood clots) [7], and the weight difference represents the blood weight [7,8]. To estimate the blood loss volume (ml), the blood weight (g) is divided by the blood density (*ρ*) [9,10]. Accurate knowledge of blood density is therefore essential, as it allows clinicians to convert weight-based measurements into reliable volume estimates, which is crucial for detecting PPH and guiding timely clinical intervention.

Blood volume comprises 40–45% red blood cells, less than 1% white blood cells and platelets, and 55% plasma [11]; these percentages may vary depending on gender, age [11], and weight or body mass index [12]. A person’s blood density is determined primarily by the red blood cells and blood volume [13]. Blood density refers to the mass per unit of blood volume [9], typically expressed in g/ml or kg/m^3^ [10]. Some textbooks indicate that human whole blood density at 37 °C is 1.060 g/ml [9,14], denser than water [14]. However, a human study found that human whole blood density ranged from 1.043 g/ml to 1.057 g/ml, which was slightly below 1.060 g/ml, but close to the reference value [15]. Aside from the conflicting evidence regarding human blood density, it is unclear whether Indonesian pregnant women’s blood density differs from that reported in the existing literature; further research is necessary to confirm these findings. Therefore, this study aimed to determine Indonesian pregnant women’s blood density to optimise the use of gravimetric method for estimating postpartum blood loss volume.

## Methods

### Study design and setting

### Participants and samples

### Data collection, handling, and analysis

#### Personnel.

In each measurement, three trained personnel were involved: a trained midwife collected blood (collector), a trained midwife measured blood density (measurer), and a trained assistant recorded and monitored the temperature (helper).

### Room setting

Blood sample collection and density measurements were conducted in adjacent sections of the same temperature-controlled room (both indoor and outdoor temperatures were monitored with a digital thermometer), separated by a half-height partition wall, to maintain procedural independence.

### Pilot

A pilot study involving three patients was conducted prior to the main study to examine the workflow and determine the optimal time period to ensure a clot-free measurement. Data from the pilot study were excluded from the final analyses.

### Sample collection

Fresh whole blood samples were collected without the addition of anticoagulants and measured immediately after collection. Each tube was coded and immediately transferred to a measurer who had no direct contact with the patients. This study used fresh whole blood without anticoagulants to obtain density values representative of native physiological conditions, thereby avoiding artefacts [16,17] arising from anticoagulant-induced changes in plasma composition, ionic strength, cellular morphology and cell count [18,19]. Immediate measurement minimises ex-vivo alterations and preserves circulating blood’s intrinsic physicochemical properties [20].

### Density measurement

Blood densities were measured using a 5 ml-pycnometer [21] following guidelines provided by the International Organisation of Vine and Wine (IOV) [22]. Before sampling, the measurer and assistant prepared all necessary equipment, including pre-weighing the empty pycnometer, to ensure a timely measurement before clot formation. The measurer filled the pycnometer with blood immediately upon receiving it, ensured there were no bubbles in it, cleaned the exterior surfaces, and weighed the filled pycnometer immediately thereafter. To ensure measurement accuracy and precision, each pycnometer was weighed three times consecutively before and after being filled with blood using a standard digital scale with a 0.001 g readability and a maximum capacity of 500 g, as recommended by the International recommendations [23] and the Indonesian Ministry of Education [24]. Blood density measurements were conducted within two minutes following its collection. The helper recorded the weighing results and ambient temperature throughout the measurement process.

### Biosafety and waste disposal

Biosafety practices were followed in all blood sample procedures. Personnel wore single-use gloves, and work surfaces were disinfected before and after each procedure. All materials that came into contact with blood were considered biohazardous waste. They were collected, sorted, and disposed of in accordance with the institution’s biosafety and waste management policies. As part of its biosafety policy, this health facility has partnered with a private company to ensure proper management and disposal of medical waste.

### Data handling and analysis

One researcher (ID) calculated blood densities, and another researcher (DA) performed statistical analyses.

### Outcomes

The primary outcome variable in this study was blood density, which was recorded on a continuous scale. The blood density assessment process involved collecting 5 ml of venous blood samples from consented participants and indirectly measuring the blood mass using a pycnometer [25], as outlined earlier.

Room temperature data were recorded on continuous scales. Maternal age, pre-pregnancy body mass index (BMI), and gestational weight gain were also collected and recorded on continuous scales. Pre-pregnancy BMI is calculated by dividing the pre-pregnancy weight (in kilograms) by the height in metres squared.

Furthermore, data on maternal age, pre-pregnancy BMI, and gestational weight gain were also grouped into clinically relevant groups based on existing literature to facilitate clinical interpretation, helping align the data analysis with real-world clinical thresholds. Maternal age was divided into three categories (i.e., under 20, between 20 and 34, and 35 or older) [26], while BMI was categorised into four categories: less than 18.5 (underweight), 18.5–22.9 (normal), 23.0–24.9 (overweight), and 25.0 or over (obese) [27]. Moreover, weight gain is classified into three categories: 9.9 or less, 10.0–14.9, and 15.0 or over [28].

### Statistical analysis

All statistical analyses were conducted using Microsoft Excel 2013 and Jamovi (version 2.6). Since blood density was measured based on a standardised protocol immediately following blood sample collection, this study had no missing data. As a result, all variables included in the analysis were complete, without imputation or case exclusion. The underlying study data are provided in Supporting Information (S2 File). Descriptive statistics were calculated to summarise the characteristics of the study variables, including mean, standard deviation, and range for continuous variables, as well as frequency and percentage for categorical variables. The normality of continuous variables was assessed using the Shapiro–Wilk test. Variables that followed a normal distribution were further analysed using parametric statistical tests, while non-normally distributed variables were analysed using non-parametric methods.

Associations between continuous variables were analysed using Pearson’s correlation. In cases where one or both variables did not meet the normality assumption, Spearman’s rank-order correlation was used instead. The threshold for statistical significance was set at p-value less than 0.05 for all tests. Moreover, to examine differences in mean blood density across categorical variables such as maternal age group, pre-pregnancy BMI categories, and gestational weight gain categories, one-way Analysis of Variance (ANOVA) was applied for variables meeting the assumptions of normality and homogeneity of variance. Post hoc analyses were performed using Tukey’s Honestly Significant Difference (HSD) test and Scheffé’s method to determine pairwise group differences. The homogeneity of variance assumption was tested using Levene’s test. For variables that violated the assumption of normality, the Kruskal–Wallis test was employed to evaluate group differences. If the Kruskal–Wallis test indicated statistically significant differences, post hoc comparisons were conducted using the Dwass–Steel–Critchlow–Fligner (DSCF) test to identify specific group pairs with significant differences.

### Ethical considerations

This study was conducted in compliance with the ethical principles outlined in the Declaration of Helsinki; the Research Ethics Committee of Universitas Hang Tuah Pekanbaru, Indonesia, approved the study protocol (Ref. No. 012/KEPK/UHTP/I/2025). All participants were provided with comprehensive information about the study’s purpose, procedures, risks, and benefits before deciding to participate; they were assured that their participation was voluntary and that they had the right to refuse to participate and to withdraw at any time (before data analysis) without penalty. All participants provided written informed consent before participating in the study. Data were anonymised immediately following collection, as each blood sample was coded before measurement, and no personally identifiable information was linked to the recorded results.

Although PPH occurs during labour, this study collected pregnant women’s blood samples rather than labouring women’s due to ethical concerns surrounding blood sample collection during labour. Obtaining blood samples during labour poses significant ethical and medical concerns due to physiological stress and potential risks associated with labour. In focusing on pregnant women, the study ensures that participants are not subjected to unnecessary stress or harm, ensuring that ethical research practices are followed, and the well-being of the participants is prioritised.

## Results

In total, 54 pregnant women who met the inclusion criteria and did not meet the exclusion criteria were invited to participate in the study; ten of them refused to participate, which resulted in 44 pregnant women being included in the analysis.

In Table 1, most participants were aged 20–34 years (86.4%) with a mean age of 27.6 years. The mean pre-pregnancy BMI was 23.8 kg/m^2^, with the largest group being normal weight (43%), followed by obese (34%). The mean gestational weight gain was 10.8 kg, and approximately 43% gained less than 10 kg. The mean and standard deviation of blood density were 1.060 ± 0.080 g/ml, ranging from 0.860 to 1.194 g/ml, with 95% CI between 1.040–1.080 g/ml. During the measurements, the indoor (mean: 27 °C, standard deviation [SD]: 1.13 °C; range: 25–30 °C) and outdoor (mean: 27 °C, SD: 1.22 °C; range: 25–30 °C) temperatures remained stable, indicating minimal thermal variability.

**Table 1:** Descriptive statistics for study variables.

Variables	n (%)
Maternal age (years) (mean ± SD, range)	27.64 ± 5.19 (17-44)
Under 20	1 (2.3)
20-34	38 (86.4)
35 or older	5 (11.4)
Pre-pregnancy body mass index^a^ (mean ± SD, range)	23.76 ± 4.09 (17.2-33.7)
Less 18.5 (Underweight)	3 (6.8)
18.5–22.9 (Normal)	19 (43.2)
23.0–24.9 (Overweight)	7 (15.9)
25.0 or over (Obese)	15 (34.1)
Gestational weight gain (kg) (mean ± SD, range)	10.82 ± 4.51 (3-22)
Less 10.0	19 (43.2)
10.0-14.9	15 (34.1)
15.0 or over	10 (22.7)
Indoor room temperature (^o^C) (mean ± SD, range)	27 ± 1.13 (25-30)
Outdoor room temperature (^o^C) (mean ± SD, range)	27 ± 1.22 (25-30)
Blood mass (g)	5.30 ± 0.40 (4.30-5.97)
Blood density (g/ml)	1.060 ± 0.080 (0.860-1.194)

^*a*^*Asia-Pacific standard* [27].

**Fig 1:**
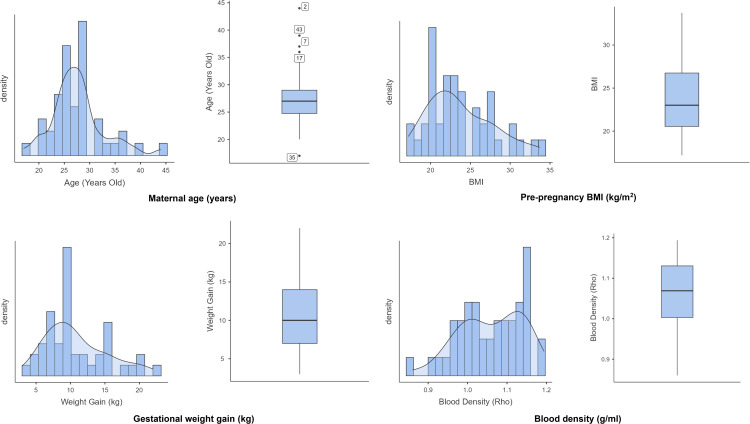
Distribution of blood density by maternal anthropometric and demographic factors. Note: Maternal age and pre-pregnancy BMI were close to normal, but slightly skewed to the right, with maternal age showing a few outliers from older women. Gestational weight gain displayed a clear right skew, indicating that most participants had lower gains, with some outliers gaining more. A symmetrical distribution of blood density was also observed, without significant outliers, indicating consistency of measurement.

Furthermore, the Shapiro-Wilk test results indicate that blood density was normally distributed (p = 0.163). Nevertheless, other variables (i.e., maternal age, pre-pregnancy BMI, and gestational weight gain) deviated significantly from normality (p < 0.05). Additionally, boxplot analysis revealed no outliers in pre-pregnancy BMI, gestational weight gain, or blood density. Despite the apparent outliers in maternal age, these values were retained for subsequent analyses as maternal age was categorised into three groups (<20 years, 20–34 years, and ≥35 years). Histograms and boxplots are provided in Fig 1 as visual representations of the distribution.

Considering the non-normal distribution of most variables, Spearman’s rank correlation coefficient was used to examine the relationship between blood density and the variables of maternal age, pre-pregnancy BMI, and gestational weight gain. Additionally, the Kruskal-Wallis test was performed to compare differences across more than two groups. If significant, the Dwass-Steel-Critchlow-Fligner (DSCF) post hoc test was applied to determine which group pairs showed significant differences.

In Table 2, Spearman’s rank correlation analysis demonstrated no significant associations between blood density and maternal age, pre-pregnancy BMI, or gestational weight gain (all p > 0.05), indicating that these maternal characteristics did not influence blood density in the study population. In contrast, a moderate positive correlation was observed between maternal age and pre-pregnancy BMI (Spearman’s rho = 0.326, p = 0.031), suggesting that pre-pregnancy BMI tended to increase with advancing maternal age. Although they were interrelated, neither factor appeared to confound blood density during pregnancy. Thus, blood density was largely independent of maternal anthropometric and demographic factors.

**Table 2:** Spearman’s rank correlation coefficient between blood density and predictor variables.

	Maternal age	Pre-pregnancy BMI	Gestational weight gain	Blood density
Maternal age	–			
Pre-pregnancy BMI	0.326 (0.031*)			
Gestational weight gain	0.140 (0.365)	0.144 (0.350)		
Blood density	0.042 (0.789)	0.162 (0.294)	−0.154 (0.318)	–

*Notes: Spearman’s rank correlation coefficients (ρ) are presented.*

* p < 0.05. ** p < 0.01.

According to a series of Kruskal–Wallis H tests, there were no statistically significant differences in blood density across maternal age groups, χ²(2) = 0.214, p = 0.899; pre-pregnancy BMI categories, χ²(3) = 1.54, p = 0.672; or gestational weight gain categories, χ²(2) = 2.52, p = 0.284. As none of the Kruskal–Wallis tests were significant, Dwass–Steel–Critchlow–Fligner (DSCF) post hoc analyses were not performed.

## Discussion

The present study assessed the blood density of Indonesian pregnant women, finding that the mean blood density at 27 °C was 1.060 g/ml (standard deviation of 0.080 g/ml), ranging from 0.860 to 1.194 g/ml. These findings are consistent with previously published data in physics and biomedical textbooks, which generally refer to a human blood density of approximately 1.060 g/ml at 37°C as a reference point [9,14]; it is denser than the mean density of water [14], which reflects the complexity of human blood composition, particularly the contribution of erythrocytes, leukocytes, thrombocytes, and plasma proteins (e.g., albumin, fibrinogen) [11], which all contribute to the overall density of blood.

Although the mean blood density in this study is consistent with existing data, the range of blood densities observed at 27 °C (0.860–1.194 g/ml) was wider than that reported at 37 °C (1.043–1.057 g/ml) [15] and slightly wider than at 23 °C (1.024–1.058 g/ml) [25]. Maternal age, pre-pregnancy BMI, and gestational weight gain were not significantly associated with blood density, indicating that additional unmeasured factors may contribute to this variability. Given the controlled environmental conditions (mean = 27 °C, SD ≈ 1 °C) and the use of a calibrated digital scale (readability: 0.001 g), device or environmental instability was unlikely to account for this variability (SD = 0.080 g/mL). Instead, biological, sample-related, and technical factors are more plausible contributors. Physiological and haematological adaptations during pregnancy may represent a key biological source of variation. Progressive plasma volume expansion, which outpaces the increase in red blood cell (RBC) mass, leads to reduced haemoglobin concentration, haematocrit, and RBC count, a well-established physiological haemodilution or dilutional anaemia [29,30], collectively lowering blood density. This process usually begins early in gestation and peaks in the late second to early third trimester but may persist until approximately 38 weeks [29,31]. In cases of true anaemia resulting from insufficient RBC mass or iron and micronutrient deficiency, the capacity for erythropoiesis is further impaired [30], accentuating haemodilution and thereby reducing blood density more markedly. Given that anaemia remains highly prevalent in low- and middle-income countries [32], including Indonesia [33], this may have contributed to the variability observed. As gestational age and haematological profiles were not recorded in the present study, these factors should be incorporated into future investigations to elucidate their joint influence on blood density. Sample-related and technical factors could also contribute to measurement dispersion. Although density determination was performed within two minutes of collection to minimise clotting, the absence of anticoagulants may still permit partial coagulation and microstructural changes, such as fibrin formation, platelet activation, and erythrocyte aggregation [34,35], which may alter the sample mass during pycnometric analysis. This highlights the methodological limitations of measuring fresh and non-anticoagulant blood. Future work should directly compare the densities obtained from anticoagulated and non-anticoagulated samples to determine the extent to which anticoagulation reduces variability. Additionally, residual air bubbles may not have been fully eliminated during sample loading, potentially introducing further errors. Improved bubble removal strategies, including degassing or more controlled filling techniques, warrant systematic evaluation.

Accurate knowledge of blood density is fundamental to interpreting gravimetric blood loss estimations, which assume a stable mass–volume relationship. Variability in blood density, whether arising from physiological changes in pregnancy, haematological conditions such as anaemia, or technical factors during sample handling, can directly affect the accuracy with which blood loss can be inferred from mass measurements. Accounting for this variability improves postpartum blood loss estimation precision. By refining a key physiological parameter in gravimetric assessment, this study reinforces the validity of a simple and scalable method for facilitating early detection of PPH, evidence-based clinical decisions, and timely intervention in PPH.

### Strengths and limitations of the study

This study has the advantage of being the first to assess blood density in pregnant women, providing novel data to the field of postpartum blood loss assessment and filling an essential gap in the literature. This contributes significantly to future research and clinical understanding of the interpretation of the gravimetric method results based on the scientific approach in estimating postpartum blood loss volume.

Despite its strengths, this study has certain limitations. Some studies recommend the use of the mechanical oscillator technique for measuring blood density due to its greater precision [13,15]; however, this method was not available in Indonesia at the time of data collection. Consequently, a conventional method, a pycnometer, was employed to measure blood density of the Indonesian pregnant women. The pycnometer is an internationally recommended [22,36] and widely used tool for measuring liquid density [37].

An error tolerance of 0.15 was adopted to enable feasible recruitment within the study timeframe. Although this may have reduced statistical precision, it was considered acceptable for a preliminary exploratory investigation. The study was further constrained by its single-site design. Nonetheless, these findings provide a practical foundation for estimating blood density and informing the refinement of gravimetric methods for postpartum blood loss assessment. Larger multisite studies with stricter error margins are warranted to enhance precision and generalisability.

In this study, ten eligible participants declined to participate, resulting in a 22.7% refusal rate, which may have introduced selection bias. Although this rate is moderate, the demographic and clinical characteristics of the included participants were comparable to those of the eligible population, indicating that significant bias was unlikely to affect the study findings. In addition, this study was limited by a single trained midwife conducting blood density measurements. While this maintained consistency and minimised inter-operator variability, it did not allow for the assessment of inter-observer reliability. In future studies, multiple measurers and devices could be employed to evaluate reproducibility and strengthen methodological robustness.

Moreover, this study did not account for gestational age or haematological parameters, limiting the interpretation of biological contributors to blood density variability. Blood density measurements were performed on fresh, non-anticoagulated samples, which may have been subjected to early coagulation and microstructural changes. Potential residual air bubbles during pycnometer filling may also introduce measurement errors. These methodological constraints should be addressed in future research.

Despite its limitations, this study provides empirical data on the density of fresh, non-anticoagulated whole blood in pregnant women, contributing to the limited body of knowledge in this under-explored domain and highlighting key methodological challenges that can guide future research.

### Generalisability

As mentioned earlier, this study was the first to evaluate the blood density of Indonesian pregnant women to improve the accuracy of interpreting gravimetric results for estimating postpartum blood loss volume. Despite providing important baseline data, the study’s findings are limited in their generalisability. The sample was drawn from one health facility, which likely represents a specific subset of pregnant Indonesian women. Due to the significant diversity in people’s characteristics in Indonesia, the findings may not apply to the entire population, which may influence the variation in blood density among people.

## Conclusion

This study indicates that the mean blood density of Indonesian pregnant women corresponds to established reference values for human blood density, providing empirical evidence to support the use of population-specific blood density values for interpreting gravimetric results in this population. Utilising this value to convert blood weight to volume enhances postpartum blood loss estimation accuracy, which enables early detection of PPH, supports evidence-based clinical decisions, and facilitates timely intervention for PPH, thereby improving maternal and labour outcomes. These findings underscore the multifactorial determinants of blood density that contribute to variations in blood density among pregnant women. To better characterise this variability, future studies should include larger and more diverse populations and systematically examine the relevant biological factors and methodological constraints, including the use of multiple devices and assessors. By establishing initial benchmarks and identifying methodological sources of error, this work lays the groundwork for more rigorous and standardised approaches in subsequent blood-density research. Additionally, future research should develop user-friendly tools for converting blood weight (g) into blood volume (ml) by adjusting population-specific blood density factors. Using such tools, care providers can more easily assess postpartum blood loss, standardise assessments across diverse populations, and ultimately improve maternal and labour outcomes.

## Supporting information

S1 File STROBE-checklist-16.12.2025. (DOC)

S2 File Supporting information data. (XLSX)
